# Efficacy of nutraceutical supplements containing *Passiflora incarnata* L., *Withania somnifera* (L.) Dunal and *Taraxacum officinale* (L.) weber ex F.H.Wigg. on markers of inflammation, oxidative stress and gut microbiota in senior dogs

**DOI:** 10.3389/fvets.2025.1695881

**Published:** 2025-11-27

**Authors:** Roberto Ciarcia, Consiglia Longobardi, Valeria Iervolino, Tommaso Manenti, Francesco Ferrucci, Iolanda Veneruso, Valeria D’Argenio, Antonio Rubino, Alessandra Pelagalli, Pietro Lombardi, Sara Damiano

**Affiliations:** 1 Department of Veterinary Medicine and Animal Production, University of Naples Federico II, Naples, Italy; 2 Biokyma Laboratories S.r.l., Arezzo, Italy; 3 CEINGE-Biotecnologie Avanzate Franco Salvatore, Naples, Italy; 4 Department of Human Sciences and Quality of Life Promotion, San Raffaele Open University, Rome, Italy; 5 Department of Advanced Biomedical Sciences, University of Naples Federico II, Naples, Italy; 6 Institute of Biostructures and Bioimages, National Research Council, Naples, Italy

**Keywords:** senior dogs, oxidative stress, inflammation, gut microbiota, medicinal plants

## Abstract

**Introduction:**

Aging in dogs is a complex phenomenon characterized by metabolic, immunological and neurological alterations that may result in chronic inflammation, oxidative stress, and intestinal microbiota changing that can compromise dogs’ overall health. Phytotherapeutics represents a promising approach to improve the quality of life in elderly dogs. In this study we have tested a mix of phytoextracts on seven old breed dogs to evaluate their synergistic beneficial effects.

**Methods:**

The mix was constituted by *Passiflora incarnata* (4% vitexin), *Withania somnifera* (2.5% withanolides), and *Taraxacum officinale* (20% inulin) administered per os for 40 days in addition to their diet. The whole blood and serum have been collected at the beginning of the experimental phase (T0), after 20 days (T1) and at the end point of the experiment, i.e., 40 days (T2), and used for biochemical analysis, detection of interleukins by ELISA, and for malondialdehyde (MDA) and Total antioxidant capacity (TAC) assay. Fresh feces have been collected at the same time points and immediately frozen to evaluate gut microbiota modification by 16S rRNA sequencing.

**Results:**

Results revealed a significant reduction of C-reactive protein (CRP) for T1 and T2 respect to the basal condition (T0) (*p* < 0.05). Moreover, a statistical decrease in IL-6 and IL-10 cytokines at both T1 and T2 was observed, probably due to mitigation of chronic inflammation in the treated old dogs. The addition of the abovementioned extracts into the diet was associated with TAC increase and reduced lipid peroxidation in the sera of aged dogs, especially at T2 (*p* < 0.01). After treatment, an interesting modulation of the microbiota was observed with a trend from T0 to T2 toward higher relative abundances of SCFA-producing taxa, although alpha and beta diversity metrics did not reach statistical significance.

**Discussion:**

This study supports the hypothesis that the integration of these herbal medicines into dietary or therapeutic regimens could represent a natural and synergistic strategy to improve the quality of life, promoting the general well-being of old dogs.

## 1 Introduction

Aging is a progressive and irreversible endogenous phenomenon that occurs through various events within the organism and leads to a reduction in strength and physical performance (1). Pet animals, particularly dogs, have emerged as an excellent model for investigating aging, as they display behavioral and physiological traits that closely resemble those of humans (2). In fact, canine models offer the opportunity to investigate conserved biological pathways and mechanisms that drive age-related decline in both species (3). Among the key hallmarks of aging, chronic low-grade systemic inflammation, often referred to as “inflammaging,” is widely recognized as a major contributing factor to the onset and progression of various age-associated disorders (4). This persistent inflammatory state is strongly linked to the pathophysiology of degenerative conditions such as osteoarthritis, cardiovascular disease, and cognitive dysfunction, highlighting the need for preventive and therapeutic strategies aimed at modulating the inflammatory response in aging individuals (4).

During aging, the inflammatory response intensifies, accompanied by the accumulation of cellular damage and the release of inflammatory mediators, including cytokines. This cascade contributes to a chronic inflammatory state, even in absence of overt inflammation. Studies in humans and dogs across various ages have demonstrated that the inflammatory process refers to the sustained upregulation of pro-inflammatory cytokines (4), such as Tumor necrosis factor-*α* (TNF-α), Interleukin-6 (IL-6) and Interleukin-1β (IL-1β). Several theories including oxidative stress (OS), inflammation, telomere shortening, mitochondrial dysfunction and misfolded proteins have been proposed to explain the mechanisms underlying aging (5). Among these, OS and inflammation are particularly prominent in aged dogs, especially in large breeds (weighing from 50 to 80 kg), which are recognized to have shorter lifespan than smaller ones (6).

OS is primarily driven by the excessive production of reactive oxygen species (ROS), which produce different free radicals, such as O_2_^−^, H_2_O_2_, capable of damaging DNA, proteins and lipids, causing the deterioration of cellular function and, ultimately, cell death. Although a basal level of ROS is essential to induce apoptosis, excessive ROS generation, often triggered by cumulative cellular damage, overwhelms the body’s antioxidant defenses, exacerbating oxidative damage (7, 8). Cells are equipped with enzymatic antioxidant systems to mitigate damage from ROS production, such as glutathione peroxidase (GPx), superoxide dismutase (SOD) and catalase (CAT). However, these defenses tend to decline with age, while oxidative damage intensifies, resulting in a redox imbalance due to a combination of increased ROS production and diminished antioxidant activity (9). Given the limitation of the endogenous antioxidant defenses, dietary antioxidant supplementation may be beneficial in preserving health and supporting immune system responses. Therefore, the inclusion of a mix of antioxidants in the diet is proposed to be beneficial through the mitigation of oxidative damage (10).

The aging process is closely associated with significant alterations in the composition of the gut microbiota, commonly referred to as dysbiosis (11). This condition is characterized by a reduction in microbial diversity and a disruption in the balance between beneficial and potentially harmful microbial populations. Such changes can negatively affect gastrointestinal function and systemic health, leading to increased intestinal permeability, persistent low-grade inflammation, and impaired immune responses in senior dogs (12). Moreover, age-related shifts in the gut microbiome have been linked to metabolic dysfunction and cognitive decline, underscoring its broad physiological relevance (12). Numerous studies have reported that aged dogs exhibit a decreased abundance of short-chain fatty acid (SCFA)-producing bacteria, such as *Faecalibacterium* and *Lactobacillus*, along with a concomitant increase in pro-inflammatory taxa, including *Clostridium perfringens* and members of the Enterobacteriaceae family (13, 14). These microbial imbalances are thought to exacerbate chronic inflammation and may contribute to the development of age-related diseases. Given the growing recognition of gut microbiota’s role in the aging process, there is increasing interest in therapeutic strategies aimed at modulating its composition. Dietary interventions, particularly those involving prebiotics, probiotics, and bioactive phytochemicals, are emerging as promising tools in veterinary medicine to support gastrointestinal health, mitigate inflammation, and promote healthy aging in dogs (15, 16). In this context, the use of synergistic combinations of natural compounds with antioxidant, anti-inflammatory, and prebiotic properties may offer enhanced benefits by acting simultaneously on multiple physiological pathways involved in the aging process. The aim of this study was to evaluate the effects of a novel dietary supplement containing natural antioxidants as an adjuvant in the prevention of oxidative stress and excessive secretion of pro-inflammatory cytokines in aged dogs. The supplement consisted of a mixed formula of *P. incarnata* (Passionflower), *W. somnifera* (Ashwagandha) and *T. officinale* (Dandelion), thanks to their anti-inflammatory, antioxidant, and prebiotic proprieties, respectively. In particular, Passionflower has long been prescribed for various indications such as anxiety, nervousness, constipation, dyspepsia, mild infections and insomnia, dysmenorrhea, epilepsy, neuralgia, but also for its sedative and analgesic effects (17, 18). Ashwagandha, also known as Indian ginseng, is an important ancient plant whose roots have been used in traditional Indian medicine systems, Ayurveda and Unani, with diuretic, narcotic, sedative and tonic activities. In particular, its use is interesting in the treatment of rheumatic pain, joint inflammation, nervous disorders and epilepsy (19, 20). The plant has also been used as a liver tonic, anti-inflammatory agent and, more recently, to treat asthma, ulcers, insomnia and senile dementia (21). Finally, in the diet of old dogs, we added Dandelion, known for both its hepatoprotective function and as a laxative and diuretic agent (22, 23). Its therapeutic activity is attributed to a variety of bioactive compounds, including polysaccharides, flavonoids, phenols, tannins, ascorbic acids, taraxol, taraerol, levulin, inulin, and luteolin. Interestingly, thanks to the oligofructans present in it, Dandelion acts also as prebiotic agent, promoting the growth of intestinal probiotics (24).

## 2 Materials and methods

### 2.1 Botanic materials

In this study a mixture of dried extract of Passionflower, Ashwagandha, and Dandelion were incorporated into the diet administered to the dogs. The extracts were provided by Biokyma srl Laboratory. The dried extract of Passionflower was obtained by extracting flowering aerial part of the herb using ethanol and water as solvent until a standard concentration of 4% of vitexin was achieved. The liquid extract was then solidified by using a spray-drying method with maltodextrin matrix. Leaves and root of Ashwagandha were processed in a similar way until a concentration of 2.5% withanolides was achieved in the dried extract. The liquid extract of Dandelion roots was obtained using water as the sole solvent, followed by spray-drying in a maltodextrin matrix until a 20% inulin content of inulin was attained.

### 2.2 Animals and treatment

Seven aged dogs (>7 years) of both sexes (four males and three females) from kennel (Dog-Kennel Service S.r.l., Nola, Italy) with no history of antibiotic treatment in the past 6 months were enrolled in the study. Inclusion criteria for the dog enrolling for the study were no antibiotic treatment in the past 6 months from the experimentation and the condition of not being pregnant or in lactation, while exclusion criteria was the obesity condition.

The average body weight of the dogs was 53 ± 2 Kg. Dogs were individually housed in indoor–outdoor runs with natural light–dark cycles and environmental enrichment. Animals had *ad libitum* access to fresh water and were maintained were continuously monitored throughout the research period and fed with a commercial adult dry food for the whole treatment. Activity was unrestricted except during sample collection periods. Each dog received an oral daily dose of 10 mg Passionflower/Kg, 10 mg of Ashwagandha /Kg, and 15 mg Dandelion /Kg of body weight (BIOKYMA- S.R.L., Italy) for 40 days. All experiments were performed in accordance with recommendations described in the Institutional Animal Care of the University of Naples Federico II and Use Committee Protocol (Protocol No. PG/2025/0026894 dated 03.03.2025). Dog blood samples were collected by veterinarians through routine blood sampling according to the guidelines of the Institutional Committee.

### 2.3 Blood, plasma and serum collection

Serum and plasma samples from all dogs were collected in Blood Collection System tubes distributed by IDEXX Laboratories (Milan, Italy). Then they were transported at refrigeration temperatures to the Pharmacology Laboratory of the Department of Veterinary Medicine and Animal Production of the University of Naples Federico II, immediately centrifuged and frozen at −80 °C until further use. For each dog, information on body mass, sex and age were collected at the time of blood sampling.

### 2.4 Biochemical analysis

C-reactive protein (CRP), glucose, Creatinine (CREA), SDMA, aspartate aminotransferase (AST), alanine aminotransferase (ALT) and Lipase were measured at three different time points: at the beginning of treatment (T0), after 20 days of treatment (T1), and at the end of treatment, i.e., 40 days post-treatment (T2). Blood was collected via the limb vein in the early morning after an overnight fast of at least 12 h. Blood aliquots were separated into a SST serum separation tube and into vacutainer blood collection tube containing lithium heparin distributed by IDEXX Laboratories (Milan, Italy). After clotting, plasma was separated by centrifuging at 3000 × *g* for 8 min in a refrigerated centrifuge, harvested, stored at −80 °C, and analyzed within 2 months. CRP, glucose, CREA, SDMA, AST, ALT, and Lipase were analyzed by IDEXX Laboratories (Milan, Italy).

### 2.5 IL-6 (interleukin-6) and IL-10 (interleukin-10) detection in serum

IL-6 and IL-10 levels were assessed in canine serum using Enzyme Linked Immunosorbent Assay (ELISA) kits (A2708 and A74431, Antibodies.com, Cambridge). For IL-6, the assay showed an intra-assay coefficient of variation (CV) < 10% and an inter-assay CV < 12%, with a dynamic range of 15.6–1,000 pg/mL. For IL-10, the intra-assay CV was <8% and the inter-assay CV < 10%, with a dynamic range of 15.625–1,000 pg/mL. Sera were diluted 1:2 with sample dilution buffer, then 100 μL were dispensed onto the plate and incubated at 37 °C. After 90 min, the liquid was removed from the plate and two washes were performed. Subsequently, 100 μL of Biotinylated Detection Antibody solution was dispensed into each well, and the plate incubated at 37 °C for 60 min. After three washing steps, 100 μL of HRP-Streptavidin Conjugate was incubated for 30 min at 37 °C, followed by other five washing. In the end, 90 μL of TMB substrate gave the colorimetric reaction, which was subsequently interrupted using the Stop solution. The colour in the wells changed from blue to yellow immediately and the OD was determined using a microplate reader (Thermo Fisher Scientific, Massachusetts) set to 450 nm. Data were analyzed according to a standard curve and expressed in pg/mL.

### 2.6 TAC (total antioxidant capacity) assay

The evaluation of oxidative stress levels in canine serum was conducted using the total antioxidant capacity (TAC) assay (ab65329, Abcam, Cambridge). This test utilises the conversion of divalent copper to monovalent copper to create a colorimetric reaction, representative of reactive oxygen species (ROS) in the examined matrix. 100 μL of serum, diluted in water at a 1:2 ratio, was plated, and 100 μL of copper solution was added. After about 40 min, the plate was analyzed using the spectrophotometer (Thermo Fisher Scientific, Massachusetts), and the data were quantified by referencing a calibration curve created with the standard (Trolox) provided in the kit. Data were expressed in Trolox equivalent capacity (mM).

### 2.7 Lipid peroxidation evaluation

Lipid peroxidation was determined following the protocol described in Da Silva Tonetto et al. (25). Briefly, 40 μL of each serum sample were combined with 20 μL of distilled water, 100 μL of acetic acid (20%), 100 μL of thiobarbituric acid (0.8%), and 40 μL od sodium dodecyl sulphate (SDS, 8%), and incubated at 100 °C. After 2 h, tubes were centrifuged and aliquots of 200 μL were transferred to a 96-well plate for spectrophotometric evaluation at 532 nm. Data were analyzed according to a calibration curve that was prepared immediately before the reading, using the malondialdehyde (MDA, Sigma, Milan, Italy) as standard and corrected by protein content, determined by Bradford assay.

### 2.8 8-Hydroxy-2-Deoxyguanosine (8OHdG) detection

DNA damage has been assessed using a Canine 8-Hydroxy-2-Deoxyguanosine (8OHdG) competitive ELISA kit (MBS743334, MyBioSource.com, San Diego, CA) on canine serum. This kit showed a inter-assay CV < 10% and a inter-assay CV < 12%, with a sensitivity of 1.0 ng/mL. Briefly, the standards and diluted samples have been incubated with 8OHdG-HRP conjugate in pre-coated plate for 1 h at 37 °C. After five washes, the plate has been incubated with HRP enzyme substrate. Finally, a stop solution has been added to the wells, and the intensity of colour has been immediately measured by a microplate reader (Thermo Fisher Scientific, Massachusetts) at 450 nm. The results have been analyzed according to an appropriate standard curve.

### 2.9 Fecal bacterial community profiling

Fecal samples were collected from all the enrolled dogs at three different time-points (T0, T1, and T2) for microbiome evaluation through 16S rRNA sequencing. All samples were immediately transferred in dry ice and within 4 h, frozen at −80 °C until analysis. Genomic DNAs were obtained using the RSC Blood DNA kit and the Maxwell RSC instrument (both from Promega, Madison, WI, USA), as previously reported (26). Two blank/negative samples were also included as controls and processed together with the dog’s samples during each analytical step to check for any potential environmental contamination. Next, 16S rRNA custom primers able to selectively amplify the V3-V4 hypervariable regions were used for microbiome composition analysis. AmpliTaq Gold polymerase, GC enhancer (both from Thermo Fisher Scientific, Waltham, MA, USA) and 20 μm of forward and reverse custom primers were used for the amplification reactions. All PCR products, verified through a 2% agarose gel, were purified by using AMPure XP beads (Beckman Coulter, Brea, CA, USA) and quality-checked on the Tape Station System with the D1000 ScreenTapes (both from Agilent Technologies, Santa Clara, CA, USA). These purified amplicons were quantified with Qubit HS (Qubit, dsDNA HS Assay, Life Technologies, Carlsbad, CA, USA), diluted to 2 ng/μL and further processed for a second-round PCR that allows NGS universal adapters ligation and samples barcoding with the Nextera DNA CD Indexes (Illumina, San Diego, CA, USA). Two additional blank/negative controls were added at each PCR step. After AMpure XP beads-based purification and Tape Station qualitative analysis, all samples, together with the six negative controls, were quantified (Qubit fluorometer), pooled in equimolar amounts and sequenced using a MiSeq system (Illumina, San Diego, CA, USA).

Bioinformatic analysis of the obtained reads in FASTQ format was then carried out using specific pipelines. Quality filtered reads were mapped against the reference database SILVA NR99 v.138 for taxonomic assignment. The obtained OTU (Operational Taxonomic Unit) and taxonomy tables were used as input files for Microbiome Analyst (version 2.0, last accession June 2025), a web-based tool supporting microbiome data analysis (27). In particular, singletons were removed for OTUs assignment and additional filtering steps included a minimum count threshold of 4, a 20% prevalence filter, and removal of constant features based on inter-quantile range (cutoff 10%). Data normalization was performed using total sum scaling (TSS).

### 2.10 Statistical analysis

The biochemical data, cytokines quantification and oxidative stress markers were analyzed by ANOVA mixed-effects analysis followed by Tukey’s multiple comparisons test or corrected for multiple comparisons by False discovery rate (FDR). α-diversity was measured by using different metrics to assess both richness and evenness and applying the ANOVA test to verify any statistically significant difference. β-diversity was evaluated by using the unweighted and weighted UniFrac distance measures with the PERMANOVA test to highlight significant differences between the study groups. Differential abundance analysis was performed using a univariate statistical test based on the EdgeR algorithm; *p*-values were corrected for multiple comparisons using the Benjamini-Hochberg False Discovery Rate (FDR). Raw 16S rRNA sequence data have been submitted to NCBI Sequence Read Archive (SRA) database under the Bio-Project accession number ID #PRJNA1333204.

## 3 Results

### 3.1 Biochemical results

Serum biochemical analysis were performed to evaluate the different physiological characteristics in older dogs at three different time points of treatment: before starting the treatment (T0), after 20 days of treatment (T1), and after 40 days of treatment (T2) (Table 1). Serum glucose, CREA, BUN, SDMA, hemoglobin reticulocyte, and albumin were within normal ranges, and no significant differences were observed before and after treatment, in all groups. A significant increase was observed in ALT, AST, Lipase, and creatine kinase levels in untreated older dogs (T0) compared to dogs in T1 and T2 groups (*p* < 0.05, *p* < 0.01). Furthermore, the level of CRP in older dogs before treatment was significantly higher compared to treated elderly dogs in T1 and T2 groups (p < 0.05).

**Table 1:** Mean of serum biochemical parameters in old dogs (*n* = 7) that received a treatment composed of a blend of dried extract of Passionflower (10 mg/Kg b.w), Ashwagandha (10 mg/Kg b.w.), and Dandelion (15 mg/Kg b.w.) daily at three different timepoints (T0 = day 0; T1 = day 20; T2 = day 40).

	T0	T1	T2
Glucose (mg/dL)	85.3 ± 6.9	83.7 ± 7.2	90.1 ± 8.9
CREA (mg/dL)	0.7 ± 0.3	0.8 ± 0.3	0.7 ± 0.2
SDMA (μg/dL)	14.7 ± 2.8	15 ± 3.9	13.8 ± 2.7
Urea-N (BUN) (mg/dL)	15.2 ± 4.3	14.6 ± 4.1	16.5 ± 2.5
Albumin (g/dL)	2.9 ± 0.4	2.8 ± 0.4	3.0 ± 0.4
ALT (U/L)	62.0 ± 38.5	30.2 ± 7.9*	32.1 ± 9.8*
AST (U/L)	75.5 ± 24.9	35.1 ± 7.3*	43.7 ± 17.4*
Lipase (U/L)	127.1 ± 12.6	95.1 ± 25.2*	98.2 ± 28.3*
CK (U/L)	275.4 ± 105.6	116.9 ± 36.7**	146.6 ± 47.5**
CRP (mg/L)	21.9 ± 15.9	11.2 ± 6.3*	9.6 ± 6.2*
Reticulocyte hemoglobin (pg)	22.8 ± 2.3	24.4 ± 2.2	23.6 ± 1.3

Reference intervals—glucose: 57.0–129.0 mg/dL; creatinine (CREA): 0.5–1.5 mg/dL; IDEXX SDMA: 0.0–14.0 μg/dL; Urea-N (BUN): 9.0–29.0 mg/dL; albumin: 2.8–4.3 g/dL; alanine aminotransferase (ALT): 25.0–122.0 U/L; aspartate aminotransferase (AST): 14.0–59.0 U/L; lipase: 0.0–250.0 U/L; Creatine kinase (CK): 41.0–378.0 U/L; C-reactive protein (CRP): 0.0–10.7 mg/L; reticulocyte hemoglobin: 24.5–31.8 pg. All blood samples were collected in the early morning after ≥12 h fasting.

Data are reported as mean ± standard deviation (SD), with all statistical comparisons performed relative to baseline group (T0); (*p < 0.05; **p < 0.01).

### 3.2 *Passiflora incarnata*, *Withania somnifera*, and *Taraxacum officinale* mix reduces pro- and anti-inflammatory cytokines in old dogs’ sera

**Figure 1:**
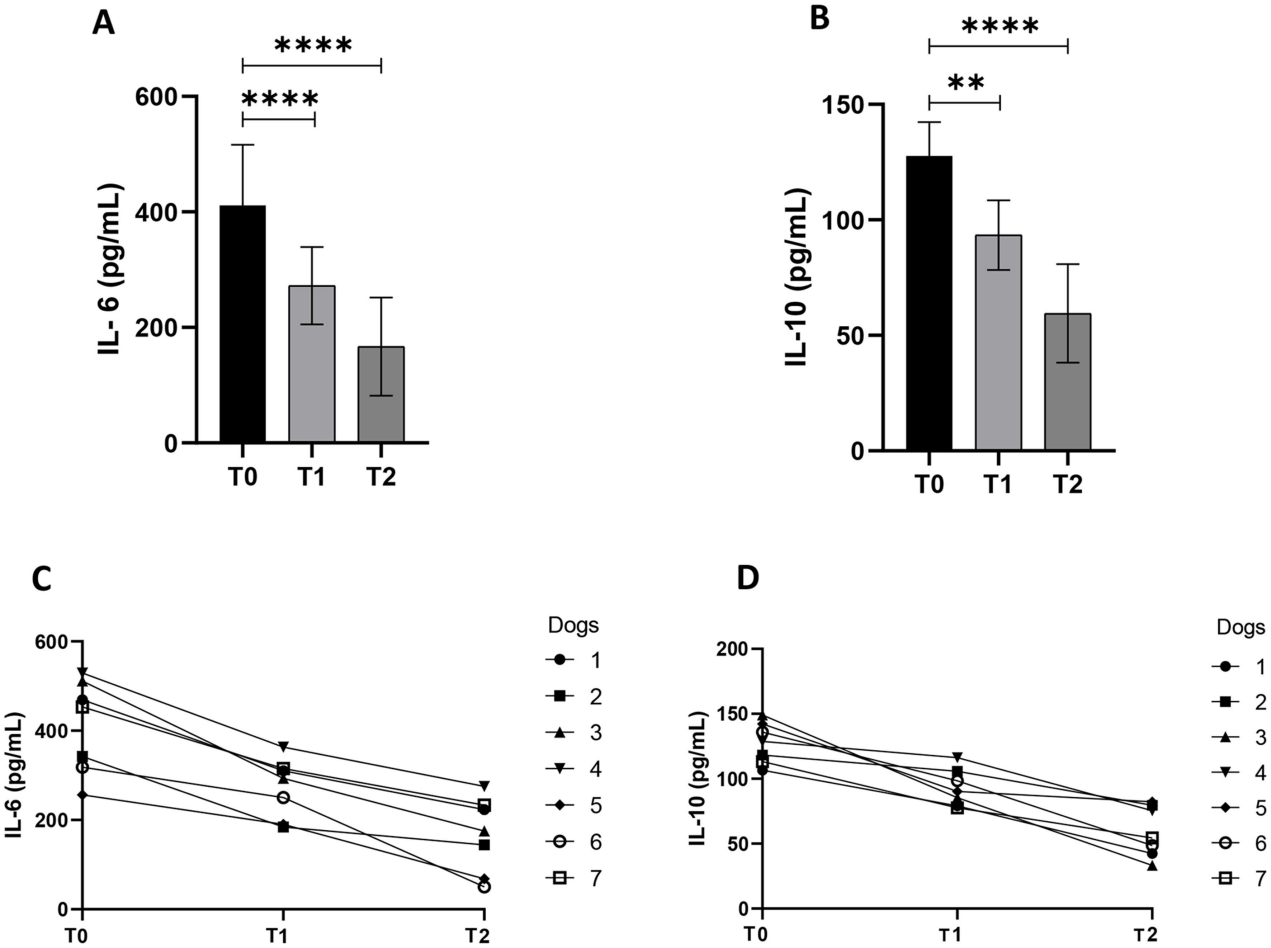
Interleukin (IL)-6 **(A)** and IL-10 **(B)** serum concentration in old dogs (*n* = 7) that received a treatment composed of a blend of dried extract of Passionflower (10 mg/Kg b.w), Ashwagandha (10 mg/Kg b.w.), and Dandelion (15 mg/Kg b.w.) daily at three different timepoints (T0 = day 0; T1 = day 20; T2 = day 40); (*p < 0.05; **p < 0.01; ****p < 0.0001). Interleukin (IL)-6 **(C)** and IL-10 **(D)** represented by spaghetti plot.

Passionflower, Ashwagandha, and Dandelion added in diet reduces pro- and anti-inflammatory cytokines in old dogs’ sera. In fact, the detection of IL-6 and IL-10 in dog’s sera showed an overall reduction in their concentrations at T1 and T2 compared to day 0 of treatment (Figure 1). IL-6 levels statistically decreased after 20 days of treatment (*p* < 0.0001; 95% CI: 86.9 to 191.0) and further decreased by the end of the treatment (*p* < 0.0001; 95% CI: 192.3 to 296.3). IL-10 exhibited the same trend: its level decreased statistically at T1 (*p* < 0.01; 95% CI: 14.8 to 54.3), reaching the lowest values at T2 (*p* < 0.0001; 95% CI: 48.6 to 88.2).

### 3.3 Daily treatment with *Passiflora incarnata, Withania somnifera,* and *Taraxacum officinale* promotes total antioxidant capacity and reduces lipid peroxidation in the sera of aged dogs

**Figure 2:**
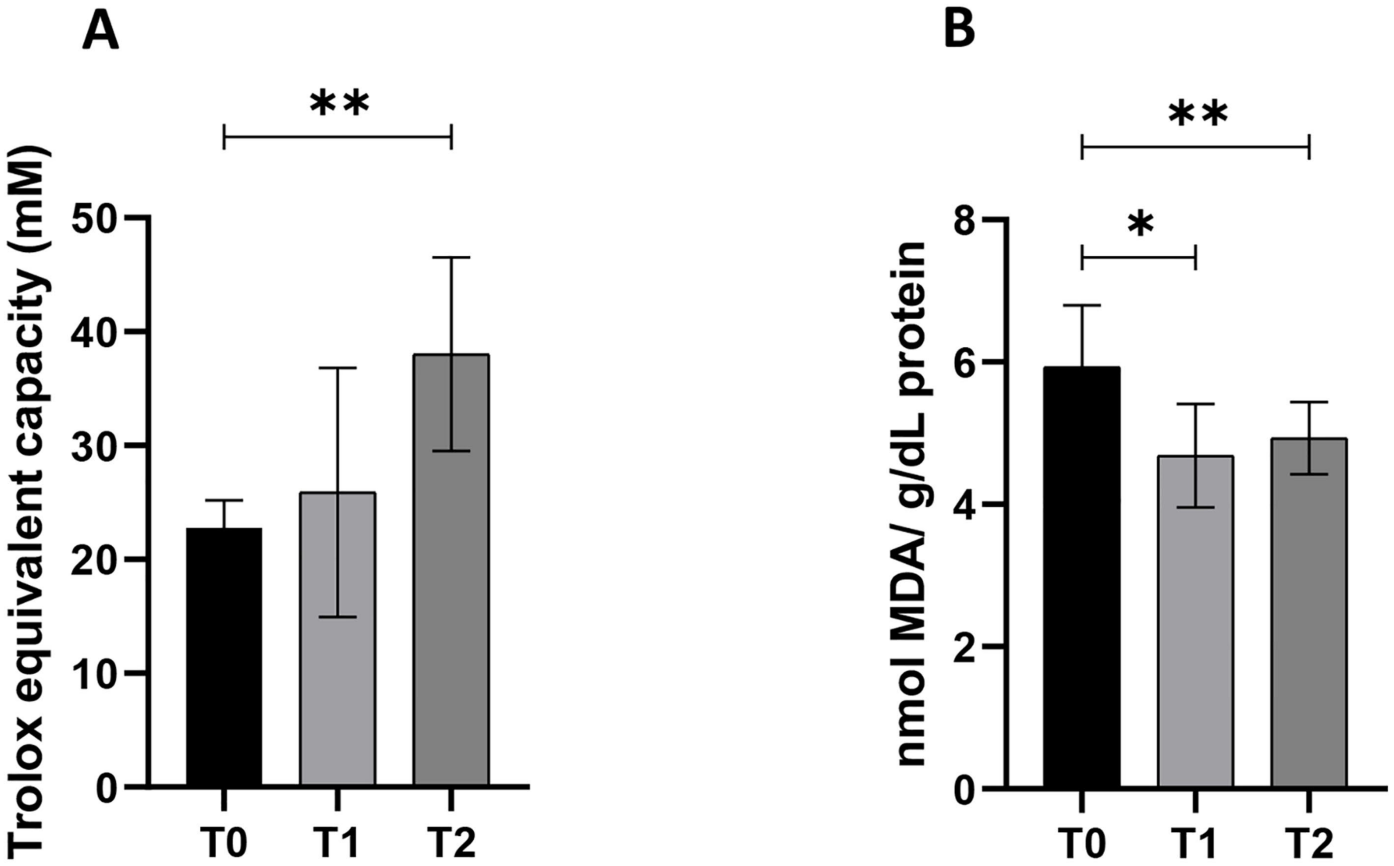
Total antioxidant capacity **(A)** and lipid peroxidation **(B)** in old dogs (*n* = 7) that received a treatment composed of a blend of dried extract of Passionflower (10 mg/Kg b.w), Ashwagandha (10 mg/Kg b.w.), and Dandelion (15 mg/Kg b.w.) daily at three different timepoints (T0 = day 0; T1 = day 20; T2 = day 40); (*p < 0.05; **p < 0.01).

The blend composed of dried extract of Passionflower, Ashwagandha, and Dandelion added in diet promotes total antioxidant capacity and reduces lipid peroxidation in the sera of aged dogs. To assess the antioxidant capability of the treatment mixture, TAC, together with MDA assay, were performed on dogs’ sera (Figure 2). TAC statistically increased at T2 (*p* < 0.01; 95% CI: −25.4 to −5.0) compared to day 0 of treatment (T0), with no statistically significant changes observed between T0 and T1. At the same time, MDA levels reduced following 20 (T1, *p* < 0.05; 95% CI: 0.3 to 2.2) and 40 days (T2, *p* < 0.01; 95% CI: 0.5 to 2.7) administration compared to the T0 timepoint, with no statistically significant changes observed between T0 and T1.

### 3.4 Mitigation of DNA damage after supplementation of passionflower, Ashwagandha, and dandelion in old dogs’ sera

**Figure 3:**
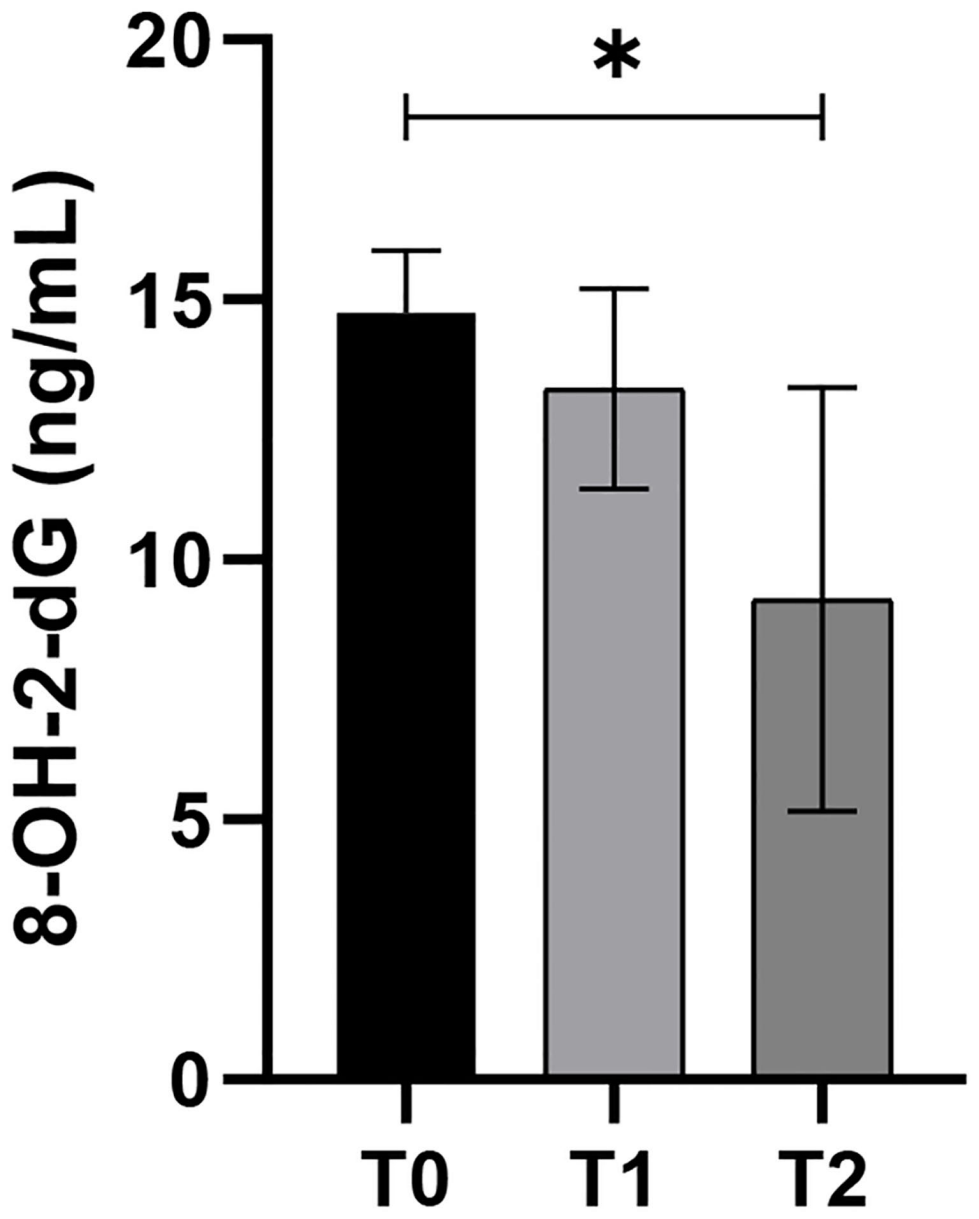
8-Hydroxy-2-Desoxyguanosine (ng/mL) in old dogs (*n* = 7) that received Passionflower (10 mg/Kg b.w), Ashwagandha (10 mg/Kg b.w.), and Dandelion (15 mg/Kg b.w.) daily at three different timepoints (T0 = day 0; T1 = day 20; T2 = day 40); (*p < 0.05).

DNA damage has been evaluated by 8-Hydroxy-2-Desoxyguanosine (8OHdG) concentration at the three time-point of the experiment. 8OHdG decreased statistically at T2 (*p* < 0.05; 95% CI: 1.4 to 9.6) compared to day 0 of treatment (T0), with no statistically significant changes observed between T0 and T1 (Figure 3).

### 3.5 Microbiome analysis results

**Figure 4:**
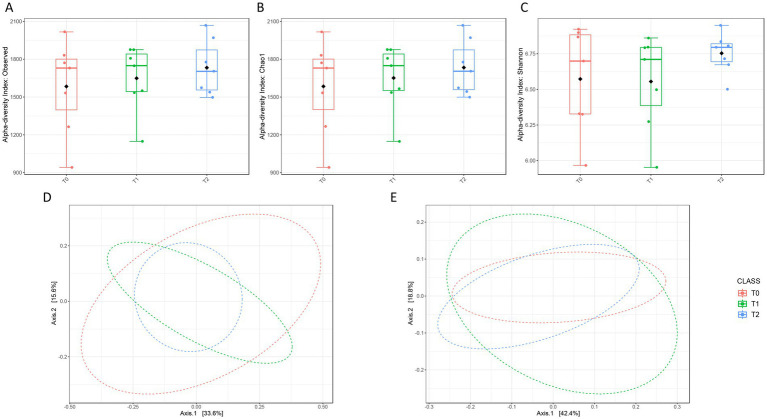
Diversity measures assessment between T0, T1, and T2. Alpha diversity has been evaluated using Observed species (**A**, *p* = 0.6), Chao1 (**B**, *p* = 0.6) and Shannon (**C**, *p* = 0.4), metrics and the ANOVA test; no significant differences were highlighted with all the used tests. Similarly, also beta diversity, assessed by using the unweighted (**D**, *p* = 0.9) and weighted (**E**, *p* = 0.3) UniFrac distance measures and applying the PERMANOVA test, resulted not significantly different between the three timepoints.

Sequencing reads were analyzed as described under Methods. All the samples were simultaneously processed and sequenced in the same sequencing run, thus minimizing potential batch effects. Interestingly, after quality filtering, no reads were assigned to the 6 negative controls, thus assessing the absence of environmental contaminations during samples processing. An average of 188,425 reads/sample (range: 136,447–244,499 reads) being equivalent to a total of 3,929 post-processing OTUs were obtained and used for the subsequent analyses. Alpha and beta diversity measures were evaluated to assess the microbial intra and inter-groups variability. In particular, alpha diversity was measured to assess richness, by using Observed Species (Figure 4A) and Chao 1 (Figure 4B) metrics, and evenness, by using Shannon index (Figure 4C). Even if no significant differences were highlighted between the three groups by ANOVA test, moving from T0 to T2 it is possible to observe a progressive increase of both richness and evenness. Beta diversity analysis also did not highlight significant differences between the three groups as assessed by both unweighted (Figure 4D) and weighted (Figure 4E) Unifrac distance measures.

**Figure 5:**
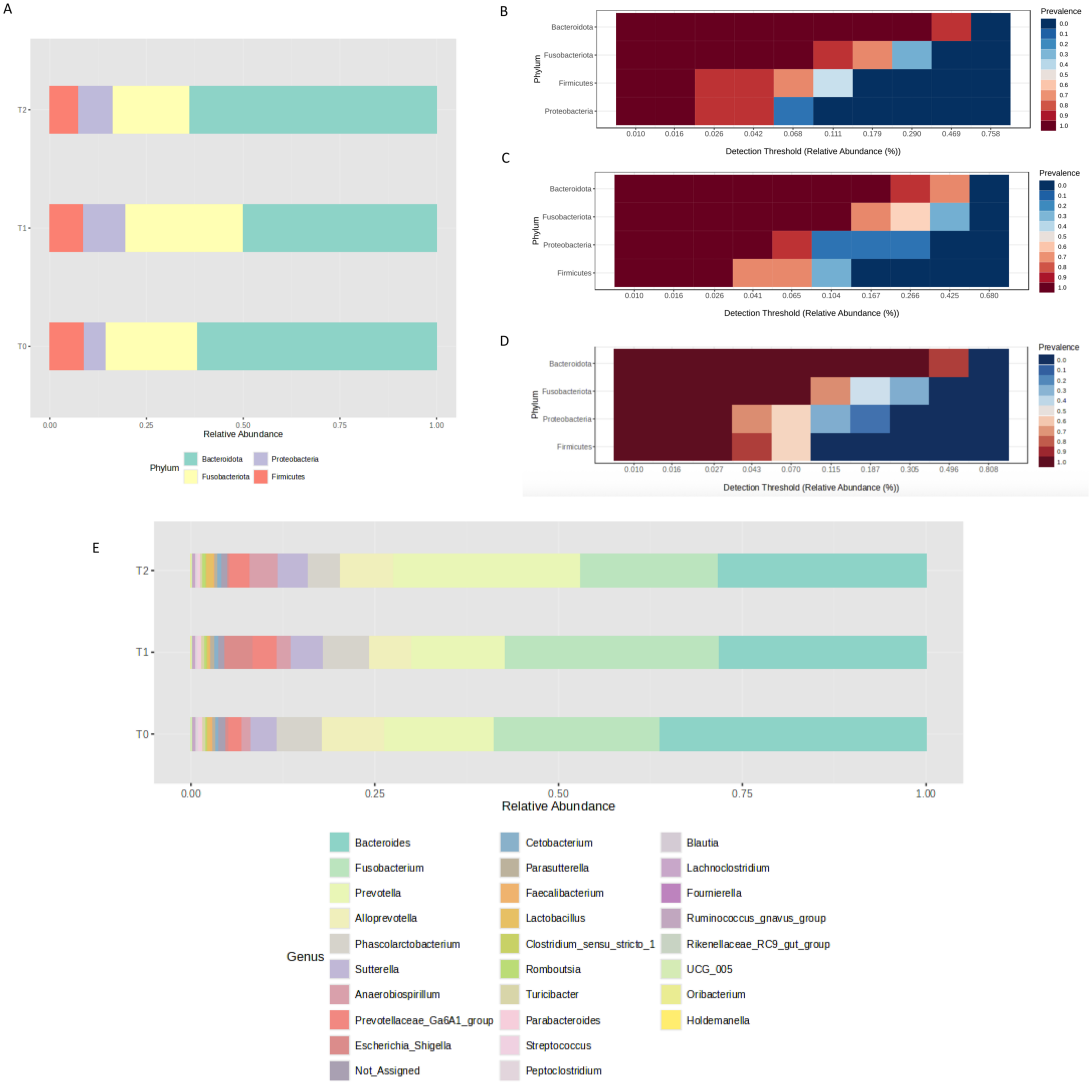
Different bacterial taxa were identified in the three tested timepoints at phylum level **(A)**, as confirmed also by core microbiome analysis highlighting different sets of bacteria contributing to the T0 **(B)**, T1 **(C)** and T2 **(D)** microbial communities. These differences were present also at genus level **(E)**.

Next, taxonomy assignment was carried out identifying a total of four phyla with a frequency >1% in all the three groups. In particular, we found that Bacteroidota was the most abundant phylum in all the tested timepoints and even if its abundance decreased at T1, it was restored at T2 (62%, 50%, and 64% of relative abundance, respectively in T0, T1 and T2). In addition, while Firmicutes relative abundance was almost stable during treatment (8.8%, 8.6%, and 7.3%), Fusobacteria (23.5%, 30.4%, and 19.8%) and Proteobacteria (5.4%, 10.7%, and 8.7%) phyla showed an increased abundance between T0 and T1 not stable at T2 where both phyla showed a reduced abundance level (Figure 5A). Accordingly, the core microbiome analysis, evaluated considering a relative abundance >1% and a 20% value of sample prevalence, allowed to identify the different taxa mainly contributing to the features of T0 (Figure 5B), T1 (Figure 5C), and T2 (Figure 5D) groups highlighting some differences between the 3 tested conditions. At genus level, 8 genera were found with a relative abundance higher than 1% in at least 1 timepoint (Figure 5E). Among these, the *Bacteroides* genus was the most abundant in all the 3 timepoints, even if it appears reduced in T1 and T2 compared with T0 (36%, 28.2%, and 28.3%). Interestingly, the *Fusobacterium* (22.5%, 29%, and 18.7%) and the *Escherichia_Shigella* (0.4%, 3.9%, and 0.4%) genera were found to have an increased abundance in the T1 group nonstable at T2, while the *Prevotella* (14.8%, 12.6%, and 25.3%) and the *Anaerobiospirillum* (1.4%, 2%, and 4%) genera showed an increased abundance at T2. Finally, the *Alloprevotella* genus showed a reduced abundance at T1 respect to T0 partially restored at T2 (8.5%, 5.8%, and 7.3%).

**Figure 6:**
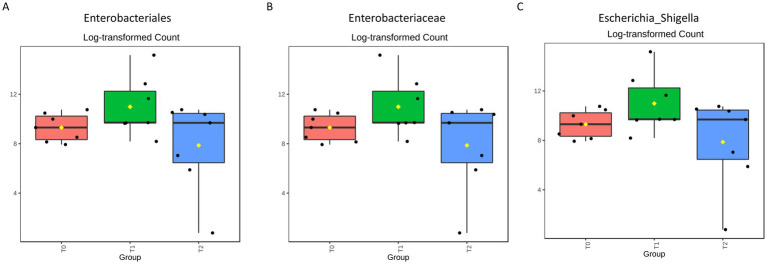
Differential abundance analysis carried out by EdgeR allowed the identification of one order (12 features totally tested) **(A)** one family (18 features totally tested) **(B)** and one genus (28 features totally tested) **(C)** significantly different between the three timepoints (*p* < 0.005 after FDR correction, using 0.05 as threshold).

Thus, to verify the presence of statistically significant differences between the three groups, differential abundance analysis was performed. No significant differences were identified between the three timepoints at phylum and class levels. However, we found that the *Enterobacteriales* order was significantly increased in the T1 respect to T2 (*p* = 0.003) (Figure 6A); within this order, the *Enterobacteriaceae* family and the *Escherichia_Shigella* genus were significantly most abundant in the T1 respect to both T0 (*p* < 0.001) and T2 (*p* < 0.001) (Figures 6B,C).

**Figure 7:**
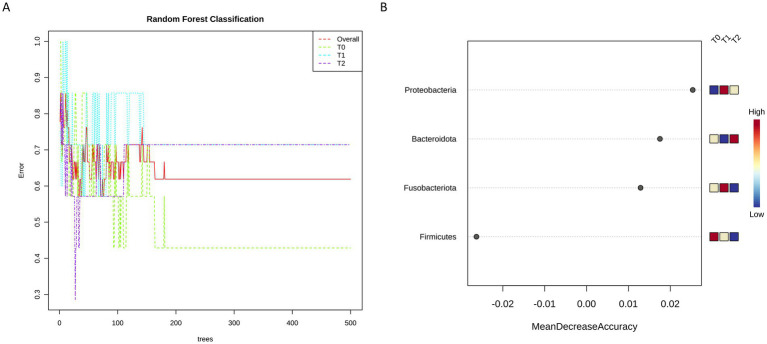
Random Forest classification allowed to identify at phylum level ta different decision tree between T0 and both Ti and T2 **(A)**, providing also the list of taxa most likely to explain this difference ranked based on their contribution **(B)**.

Finally, Random Forest analysis was carried out to verify whether the bacterial microbial composition could discriminate among the three tested timepoints. Interestingly, we found that at phylum level the obtained decision tree clearly separates T0 respect to T1 and T2 (Figure 7A), also providing a list of taxa mainly contributing to this difference (Figure 7B).

## 4 Discussion

In recent years, a significant increase in longevity has been observed in both humans and pets. However, longevity has been accompanied by the development of morphological and functional changes throughout life, resulting in an increase in age-related injuries and diseases (25, 26). Because pets share their owners’ environment and are therefore exposed to the same environmental factors and pathological stimuli, they can develop several spontaneous diseases comparable to those in humans. For this reason, this research falls within a translational research framework (27).

In this research, the possible therapeutic effect of a mix of medicinal plants extract of Passionflower, Ashwagandha, and Dandelion on the improvement of biochemical, anti-inflammatory and oxidative parameters of the senior dog was evaluated. Passionflower, in particular the dried aerial part of the plant, is officially included in several national pharmacopoeias and its interest in clinical use has increased in the last decade (17, 18). Due to an increasing number of favorable data cited in the media and in many countries, these products are often labeled as natural food supplements (17). Moreover, the pharmacological activities of Ashwagandha, attributed to the roots of the plant and to the steroid lactones present in it, including choline, tropanol, pseudotopanol, cuscokygrene, 3- tigioyloxytropane, and isopelletierin were also included in many national pharmacopoeias. Finally, in the diet of old dogs, Dandelion was also added to the diet because several studies conducted in mice have shown its efficacy against the cytotoxic effects of ethanol and sodium dichromate by improving liver enzymes associated with the restoration of liver cell histopathology (24).

Given their anti-inflammatory, calming, and prebiotic properties, we have decided to test the effects of Passionflower, Ashwagandha, and Dandelion supplementation in standard diet of old dog with arthritis and liver disease. In particular, we have analyzed the biochemical parameters related to the two organs mainly involved in metabolism, kidney and liver, inflammation and OS because of their well-known involvement in aging and in the quality of life of the elderly patient (2, 28).

Although the use of natural substances is appreciated by consumers as they are considered healthy and presumed lack of toxic effects, further studies are needed to verify their safety. Current safety assessment of these natural ingredients is often limited to documented history of human clinical data, which may not adequately reflect species-specific responses. Therefore, improved data collection on potential clinical and toxicological effects in targeted species are warranted. It is important to consider organ-specific toxicity, particularly the liver, the major metabolic organ, and to a lesser extent, the kidneys as key excretory organ. In this work we focused our attention on the values of AST, ALT, and Lipase, known as the main markers of liver function. It was interesting to note that the mix of natural plants did not induce liver damage but appeared to improve hepatic functionality over time. Furthermore, renal function parameters, such as SDMA and CREA, did not undergo functional alterations. Overall, these findings indicate a favorable safety of the mix administered orally and suggest a possible hepatoprotective effect, mainly attributable to dandelion (24). The significant decline in CK values observed throughout the study period, although remaining within the reference range, may indicate a positive impact of the botanical mixture also on musculoskeletal health or a reduction in muscle micro-damage. In fact, elevated AST and CK serum activities with ALT within the reference interval suggest muscle injury, as reported by Oikonomidis and Milne (29). Nonetheless, CK exhibits significant sensitivity to pre-analytical variables, and such pronounced fluctuations may indicate handling stress or recent physical activity, rather than a genuine therapeutic effect (29).

OS is detectable in several pathological states and involves an increase in cellular reactive oxygen species (ROS) levels and the decrease of the antioxidant capacity of the cell, creating damage at the level of nucleic acids, proteins and lipids (30). ROS are linked to the bioactivation of the drug to hepatotoxicity and as a direct mechanistic indicator of a compound’s hepatotoxic potential (31). Dogs enrolled in this trial showed, before the treatment, lipid peroxidation value higher respect to treated groups. In fact, this value decreased significantly and time-dependently at the T1 and T2 groups, demonstrating an important protective effect on oxidative stress. In normal aging, it is well demonstrated that mitochondrial respiratory chain activity declines and the rate of somatic mitochondrial DNA mutations increases and thus, combined with lower endogenous antioxidant activity, may lead to increasing oxidative damage (32). In accordance with previous authors, (30, 33) we observed in old untreated dogs an important alteration of redox status, measured by the increase of lipid peroxidation, and a decrease in antioxidant status, measured through TAC assay. It is interesting to note that the mix of Passionflower, Ashwagandha, and Dandelion in standard diet is able to restore the oxidant/antioxidant status. This effect could be ascribed to the Withaferin A of Ashwagandha. In fact, it may contribute to Nrf2 transcription factor activation, which modulates the expression of endogenous antioxidants, contributing to the OS reduction in old dogs’ sera after treatment (34).

The role of ROS in biology is closely related to inflammation (35). During the inflammatory process, the production of superoxide by NADPH oxidase in the cell leads to the formation of harmful ROS. With increasing age, the release of proinflammatory cytokines in both humans and dogs leads to a significant increase in acute phase proteins in circulation, such as CRP (36). Furthermore, the measurement of CRP has some advantages over that of cytokines as it remains more stable for prolonged periods of time and its synthesis speed is determined by the intensity of the inflammatory process (36). In this study a significant decrease was observed in CRP (Table 1), demonstrating how the mix of plants used as food additive was able, in a very significant way, to reduce this important marker of systemic inflammation. These findings are in accordance with other studies using other herbal medicine with anti-inflammatory properties (37). CRP reduction may hypothetically be related to the modulation of Nuclear factor kappa b (NF-κB). Phytochemicals present in the administered botanicals, including withanolides from *W. somnifera*, flavonoids from *P. incarnata*, and sesquiterpene lactones from *T. officinale*, have been reported to inhibit NF-κB activation by preventing its nuclear translocation and subsequent transcriptional activity (38–41). Additionally, the observed decrease in IL-6 levels may indicate not only a direct anti-inflammatory effect of the treatment but also an enhancement of redox homeostasis, likely through the reduction of oxidative stress and the restoration of antioxidant defenses (42), in accordance to our findings. Interestingly, the mix showed a decrease in both pro-inflammatory IL-6 and anti-inflammatory IL-10, reflecting an immunomodulatory response, more than an anti-inflammatory one. IL-10 production often rises during active inflammation as a compensatory mechanism to counteract excess in pro-inflammatory signaling. The observed decrease in IL-10 could indicate a resolution of the inflammatory process, characterized by a physiological rebalancing of pro- and anti-inflammatory mediators. Indeed, phytoextracts are known to influence immune responses through a complex network of biochemical pathways, contributing to the immunomodulation of the immune system (43).

DNA damage is known to be present during inflammation and has been shown to induce inflammation both *in vitro* and *in vivo* (44). Its epigenetic role as a hallmark of aging makes it noteworthy (45). In fact, OS can cause DNA damage by producing the tautomeric 8OHdG, resulting in DNA mutations, often associated with premature aging syndromes (9). In our treated older dogs, the significant reduction in 8OHdG aligns with findings from studies on canine models, where antioxidant supplements have been shown to attenuate oxidative DNA damage. For instance, Heaton et al. reported that dietary supplementation containing vitamin E, taurine, and carotenoids in adult dogs significantly reduced endogenous and exogenous DNA damage, concomitantly with an increase in TAC (46). Therefore, the observed parallel reduction in OS and DNA damage in our study suggests a protective effect mediated by the antioxidant compounds present in the phytocomplex.

In adult and senior dogs, the gut microbiota exhibits a high degree of resilience and remains relatively stable over time, often resisting perturbations induced by dietary or nutraceutical interventions. Previous studies in aged canines have shown that alpha diversity, reflecting species richness and evenness, remains largely unchanged unless interventions are both intensive and prolonged (47). Consistently with these findings, our treatment produced no statistically changes in alpha and beta diversity. It is plausible that the phytocompounds in our mix exerted primarily functional effects, such as altering microbial metabolites, stimulating SCFA production, or reducing oxidative intestinal stress, rather than causing significant structural shifts in the bacterial community. Indeed, dietary interventions capable of inducing substantial diversity changes typically span durations ≥8 weeks or involve stronger perturbations (48). Our 40-day treatment may thus have fallen short of the threshold necessary to elicit global changes in diversity metrics. This may explain why no significant differences were detected in alpha or beta diversity overall. Furthermore, age-associated phenomena such as immunosenescence and chronic low-grade inflammation (“inflammaging”) likely diminish the intestinal ecosystem’s capacity to respond to external stimuli, further limiting its plasticity (49). These observations are corroborated by recent large-scale investigations in senior dogs, which confirm that while certain taxa shift with age, overall diversity and community structure remain stable (50). The transient increase in *Enterobacteriales*, specifically *Escherichia_Shigella*, at T1 may indicate an initial adaptive response of the gut microbiota to the nutraceutical intervention. This phenomenon has been observed in dietary intervention studies, where elevated levels of Proteobacteria, including *Enterobacteriales*, were identified shortly after the introduction of new dietary components, but normalised over time as the microbiota stabilised (48). These transient blooms may signify a phase of ecological reorganisation within the gut ecosystem prior to achieving a new homeostatic state. After treatment, dogs resulted in a small, non-statistical increase in *Fusobacterium* and *Firmicutes*, which normally co-dominates the fecal microbiota alongside *Bacteroidetes*, accounting for approximately 20%–30% of the bacterial community in healthy adult dogs (51). This contrasts with the human gut, where *Fusobacterium* is frequently associated with gastrointestinal pathologies, such as colorectal cancer and inflammatory bowel disease (52). In canids, however, it appears to play a beneficial role, being positively correlated with enteric health and actively involved in protein metabolism and SCFA production, including butyrate, an important substrate for colonocytes health (47). These effects may be attributable to the *Taraxacum* of the mix.

The short length of this study and small sample size are two major issues. Therefore, subsequent studies with larger cohorts and extended follow-up durations are essential to validate these preliminary observations. Our findings suggest a potential correlation between nutraceutical treatment and changes in inflammation, oxidative stress markers and gut health in aging dogs. The absence of a diet-matched or placebo control group necessitates that the results be regarded as exploratory evidence rather than conclusive proof of causality. Randomisation and blinding can mitigate certain biases; however, temporal and kennel-related factors may have influenced the observed effects. Subsequent studies utilising control cohorts or crossover designs are anticipated to replicate and expand upon these preliminary findings. Moreover, with regard to microbiome analysis, alternative compositional approaches (e.g., ANCOM-BC or DESeq2 with CLR-transformed data) may provide complementary insights in further studies on enlarged cohorts.

## 5 Conclusion

In conclusion, this exploratory study offers initial evidence that dietary supplementation with a standardised blend of *P. incarnata*, *W. somnifera*, and *T. officinale* may correlate with beneficial trends in inflammatory, oxidative stress, and biochemical markers in senior dogs, without inducing significant alterations in overall gut microbial diversity. These findings bolster the hypothesis that botanical nutraceuticals, in synergy, may play a role in preserving health and enhancing the quality of life in aging dogs.

## Data Availability

The microbiome data presented in the study are deposited in the NCBI Sequence Read Archive (SRA) database, accession number ID #PRJNA1333204.

## References

[1] da Costa JP Vitorino R Silva GM Vogel C Duarte AC Rocha-Santos T. A synopsis on aging—theories, mechanisms and future prospects. Ageing Res Rev. (2016) 29:90–112. doi: 10.1016/j.arr.2016.06.005, PMID: 27353257 PMC5991498

[2] Hoffman JM Creevy KE Franks A O’Neill DG Promislow DEL. The companion dog as a model for human aging and mortality. Aging Cell. (2018) 17:e12737. doi: 10.1111/acel.12737 29457329 PMC5946068

[3] Cohen AA. Aging across the tree of life: the importance of a comparative perspective for the use of animal models in aging. Biochim Biophys Acta Mol basis Dis. (2018) 1864:2680–9. doi: 10.1016/j.bbadis.2017.05.028, PMID: 28690188

[4] Franceschi C Campisi J. Chronic inflammation (Inflammaging) and its potential contribution to age-associated diseases. J Gerontol A Biol Sci Med Sci. (2014) 69:S4–9. doi: 10.1093/gerona/glu057, PMID: 24833586

[5] Cohen AA. Complex systems dynamics in aging: new evidence, continuing questions. Biogerontology. (2016) 17:205–20. doi: 10.1007/s10522-015-9584-x, PMID: 25991473 PMC4723638

[6] Michell AR. Longevity of British breeds of dog and its relationships with sex, size, cardiovascular variables and disease. Vet Rec. (1999) 145:625–9. doi: 10.1136/vr.145.22.625, PMID: 10619607

[7] Dowling DK Simmons LW. Reactive oxygen species as universal constraints in life-history evolution. Proc R Soc B Biol Sci. (2009) 276:1737–45. doi: 10.1098/rspb.2008.1791, PMID: 19324792 PMC2674489

[8] Monaghan P Metcalfe NB Torres R. Oxidative stress as a mediator of life history trade-offs: mechanisms, measurements and interpretation. Ecol Lett. (2009) 12:75–92. doi: 10.1111/j.1461-0248.2008.01258.x, PMID: 19016828

[9] Hasty P Campisi J Hoeijmakers J Van Steeg H Vijg J. Aging and genome maintenance: lessons from the mouse? Science (2003) 299: 1355–1359 doi: 10.1126/science.1079161 12610296

[10] Panickar KS Jewell DE. The beneficial role of anti-inflammatory dietary ingredients in attenuating markers of chronic low-grade inflammation in aging. Horm Mol Biol Clin Investig. (2015) 23:59–70. doi: 10.1515/hmbci-2015-0017, PMID: 26124060

[11] AlShawaqfeh MK Wajid B Minamoto Y Markel M Lidbury JA Steiner JM . A dysbiosis index to assess microbial changes in fecal samples of dogs with chronic inflammatory enteropathy. FEMS Microbiol Ecol. (2017) 93:fix136. doi: 10.1093/femsec/fix136 29040443

[12] Mondo E Marliani G Accorsi PA Cocchi M Di Leone A. Role of gut microbiota in dog and cat’s health and diseases. Open Vet J. (2019) 9:253–8. doi: 10.4314/ovj.v9i3.10, PMID: 31998619 PMC6794400

[13] Templeton GB Fefer G Case BC Roach J Azcarate-Peril MA Gruen ME . Longitudinal analysis of canine oral microbiome using whole genome sequencing in aging companion dogs. Animals. (2023) 13:3846. doi: 10.3390/ani13243846, PMID: 38136883 PMC10740535

[14] Balouei F Rivera C de Paradis A Stefanon B Kelly S McCarthy N . Gut microbiota variation in aging dogs with osteoarthritis Animals 2025 15:1619 doi: 10.3390/ani15111619 40509085 PMC12153520

[15] Norouzkhani N Afshari S Sadatmadani SF Mollaqasem MM Mosadeghi S Ghadri H . Therapeutic potential of berries in age-related neurological disorders. Front Pharmacol. (2024) 15:1348127. doi: 10.3389/fphar.2024.1348127, PMID: 38783949 PMC11112503

[16] Iriondo-DeHond A Martorell P Genovés S Ramón D Stamatakis K Fresno M . Coffee silverskin extract protects against accelerated aging caused by oxidative agents. Molecules. (2016) 21:21. doi: 10.3390/molecules21060721, PMID: 27258247 PMC6274150

[17] Miroddi M Calapai G Navarra M Minciullo PL Gangemi S. *Passiflora incarnata* L.: ethnopharmacology, clinical application, safety and evaluation of clinical trials. J Ethnopharmacol. (2013) 150:791–804. doi: 10.1016/j.jep.2013.09.047, PMID: 24140586

[18] Rodriguez-Fragoso L Reyes-Esparza J Burchiel S Herrera-Ruiz D Torres E. Risks and benefits of commonly used herbal medicines in México. Toxicol Appl Pharmacol. (2008) 227:125–35. doi: 10.1016/j.taap.2007.10.005 18037151 PMC2322858

[19] Speers AB Cabey KA Soumyanath A Wright KM. Effects of *Withania somnifera* (Ashwagandha) on stress and the stress- related neuropsychiatric disorders anxiety, depression, and insomnia. Curr Neuropharmacol. (2021) 19:1468–95. doi: 10.2174/1570159x19666210712151556, PMID: 34254920 PMC8762185

[20] Mandlik DS Namdeo AG. Pharmacological evaluation of Ashwagandha highlighting its healthcare claims, safety, and toxicity aspects. J Diet Suppl. (2021) 18:183–226. doi: 10.1080/19390211.2020.1741484 32242751

[21] Sultana N Choudhury Shimmi S Tanveer M Parash H Akhtar J. Effects of ashwagandha (*Withania somnifera*) root extract on some serum liver marker enzymes (AST, ALT) in gentamicin intoxicated rats. J Bangladesh Soc Physiol. (2012) 7:1–7. doi: 10.3329/jbsp.v7i1.11152

[22] You Y Yoo S Yoon HG Park J Lee YH Kim S . In vitro and in vivo hepatoprotective effects of the aqueous extract from *Taraxacum officinale* (dandelion) root against alcohol-induced oxidative stress. Food Chem Toxicol. (2010) 48:1632–7. doi: 10.1016/j.fct.2010.03.037, PMID: 20347918

[23] Rohilla R Garg T Goyal AK Rath G. Herbal and polymeric approaches for liver-targeting drug delivery: novel strategies and their significance. Drug Deliv. (2016) 23:1–17. doi: 10.3109/10717544.2014.945018, PMID: 25101832

[24] Mahboubi M Mahboubi M. Hepatoprotection by dandelion (*Taraxacum officinale*) and mechanisms. Asian Pac J Trop Biomed. (2020) 10:1–10. doi: 10.4103/2221-1691.273081

[25] Dias-Pereira P. Morbidity and mortality in elderly dogs – a model for human aging. BMC Vet Res. (2022) 18:457. doi: 10.1186/s12917-022-03518-8, PMID: 36581919 PMC9798575

[26] Chapagain D Range F Huber L Virányi Z. Cognitive Aging in Dogs. Gerontology. (2018) 64:165–71. doi: 10.1159/000481621, PMID: 29065419 PMC5841136

[27] Creevy KE Akey JM Kaeberlein M Promislow DEL Barnett BG Benton B . An open science study of ageing in companion dogs. Nature. (2022) 602:51–7. doi: 10.1038/s41586-021-04282-9, PMID: 35110758 PMC8940555

[28] Sándor S Kubinyi E. Genetic pathways of aging and their relevance in the dog as a natural model of human aging. Front Genet. (2019) 10:948. doi: 10.3389/fgene.2019.00948, PMID: 31681409 PMC6813227

[29] Oikonomidis IL Milne E. Clinical enzymology of the dog and cat. Aust Vet J. (2023) 101:465–78. doi: 10.1111/avj.13291, PMID: 37767749

[30] Sies H Belousov VV Chandel NS Davies MJ Jones DP Mann GE . Defining roles of specific reactive oxygen species (ROS) in cell biology and physiology. Nat Rev Mol Cell Biol. (2022) 23:499–515. doi: 10.1038/s41580-022-00456-z, PMID: 35190722

[31] Shuhendler AJ Pu K Cui L Uetrecht JP Rao J. Real-time imaging of oxidative and nitrosative stress in the liver of live animals for drug-toxicity testing. Nat Biotechnol. (2014) 32:373–80. doi: 10.1038/nbt.2838, PMID: 24658645 PMC4070437

[32] Wallace DC. Mitochondrial genetics: a paradigm for aging and degenerative diseases? Science. 256:628–32. doi: 10.1126/science.1533953, PMID: 1533953

[33] Choi K Ortega MT Jeffery B Riviere JE Monteiro-Riviere NA. Oxidative stress response in canine in vitro liver, kidney and intestinal models with seven potential dietary ingredients. Toxicol Lett. (2016) 241:49–59. doi: 10.1016/j.toxlet.2015.11.012, PMID: 26602166

[34] Grace YS Runting L Jiankun C Mark H Zezong G Kevin LF . *Withania somnifera* and its withanolides attenuate oxidative and inflammatory responses and up-regulate antioxidant responses in BV-2 microglial cells. NeuroMolecular Med. (2016) 18:241–52. doi: 10.1007/s12017-016-8411-0 27209361

[35] Murphy MP Holmgren A Larsson NG Halliwell B Chang CJ Kalyanaraman B . Unraveling the biological roles of reactive oxygen species. Cell Metab. (2011) 13:361–6. doi: 10.1016/j.cmet.2011.03.010, PMID: 21459321 PMC4445605

[36] Giovannini S Onder G Liperoti R Russo A Carter C Capoluongo E . Interleukin-6, C-reactive protein, and tumor necrosis factor-alpha as predictors of mortality in frail, community-living elderly individuals. J Am Geriatr Soc. (2011) 59:1679–85. doi: 10.1111/j.1532-5415.2011.03570.x, PMID: 21883115 PMC4321727

[37] Yatoo M. Anti-inflammatory drugs and herbs with special emphasis on herbal medicines for countering inflammatory diseases and disorders - a review. Recent Patents Inflamm Allergy Drug Discov. (2018) 12:39–58. doi: 10.2174/1872213X12666180115153635, PMID: 29336271

[38] Singh A Raza A Amin S Damodaran C Sharma AK. Recent advances in the chemistry and therapeutic evaluation of naturally occurring and synthetic Withanolides. Molecules. (2022) 27:886. doi: 10.3390/molecules27030886, PMID: 35164150 PMC8840339

[39] Ichikawa H Takada Y Shishodia S Jayaprakasam B Nair MG Aggarwal BB. Withanolides potentiate apoptosis, inhibit invasion, and abolish osteoclastogenesis through suppression of nuclear factor-ΚB (NF-ΚB) activation and NF-ΚB-regulated gene expression. Mol Cancer Ther. (2006) 5:1434–45. doi: 10.1158/1535-7163.MCT-06-0096, PMID: 16818501

[40] Chun JK Seo D-W Ahn SH Park JH You J-S Lee C-H . Suppression of the NF- k B signalling pathway by ergolide, sesquiterpene lactone, in HeLa cells. J Pharm Pharmacol. (2007) 59:561–6. doi: 10.1211/jpp.59.4.0011, PMID: 17430640

[41] Guo S Rezaei MJ. The benefits of ashwagandha (*Withania somnifera*) supplements on brain function and sports performance. Front Nutr. (2024) 11:1439294. doi: 10.3389/fnut.2024.1439294, PMID: 39155932 PMC11327513

[42] Didion SP. Cellular and oxidative mechanisms associated with interleukin-6 signaling in the vasculature. Int J Mol Sci. (2017) 18:2563. doi: 10.3390/ijms18122563, PMID: 29186034 PMC5751166

[43] Shukla MK Singh SK Pandey S Gupta PK Choudhary A Jindal DK . Potential immunomodulatory activities of plant products. S Afr J Bot. (2022):937–43. doi: 10.1016/j.sajb.2022.04.055

[44] Chatterjee N Walker GC. Mechanisms of DNA damage, repair, and mutagenesis. Environ Mol Mutagen. (2017) 58:235–63. doi: 10.1002/em.22087, PMID: 28485537 PMC5474181

[45] Siametis A Niotis G Garinis GA. DNA damage and the aging epigenome. J Invest Dermatol. (2021) 141:961–7. doi: 10.1016/j.jid.2020.10.006, PMID: 33494932

[46] Heaton PR Reed CF Mann SJ Ransley R Stevenson J Charlton CJ . Role of dietary antioxidants to protect against DNA damage in adult dogs. J Nutr. (2002) 132:1720–4. doi: 10.1093/jn/132.6.1720S 12042506

[47] Pilla R Suchodolski JS. The role of the canine gut microbiome and metabolome in health and gastrointestinal disease. Front Vet Sci. (2020) 6:498. doi: 10.3389/fvets.2019.00498, PMID: 31993446 PMC6971114

[48] Schmidt M Unterer S Suchodolski JS Honneffer JB Guard BC Lidbury JA . The fecal microbiome and metabolome differs between dogs fed bones and raw food (BARF) diets and dogs fed commercial diets. PLoS One. (2018) 13:e0201279. doi: 10.1371/journal.pone.0201279, PMID: 30110340 PMC6093636

[49] Shin NR Whon TW Bae JW. Proteobacteria: microbial signature of dysbiosis in gut microbiota. Trends Biotechnol. (2015) 33:496–503. doi: 10.1016/j.tibtech.2015.06.011, PMID: 26210164

[50] Fernández-Pinteño A Pilla R Manteca X Suchodolski J Torre C Salas-Mani A. Age-associated changes in intestinal health biomarkers in dogs. Front Vet Sci. (2023) 10:1213287. doi: 10.3389/fvets.2023.1213287, PMID: 37680388 PMC10481537

[51] Garrigues Q Apper E Chastant S Mila H. Gut microbiota development in the growing dog: a dynamic process influenced by maternal, environmental and host factors. Front Vet Sci. (2022) 9:964649. doi: 10.3389/fvets.2022.964649 36118341 PMC9478664

[52] Gethings-Behncke C Coleman HG Jordao HWT Longley DB Crawford N Murray LJ . *Fusobacterium nucleatum* in the colorectum and its association with cancer risk and survival: a systematic review and meta-analysis. Cancer Epidemiol Biomarkers Prev. (2020) 29:539–48. doi: 10.1158/1055-9965.EPI-18-1295, PMID: 31915144

